# Radiological Progression of Degenerative Cervical Myelopathy in a Clinically Stable Patient: Case Report

**DOI:** 10.2196/48212

**Published:** 2024-06-27

**Authors:** Rishi Umeria, Oliver Mowforth, Munashe Veremu, Benjamin Davies, Mark Kotter

**Affiliations:** 1 Division of Neurosurgery Department of Clinical Neurosciences University of Cambridge Cambridge United Kingdom

**Keywords:** degenerative cervical myelopathy, neurosurgery, radiology, magnetic resonance imaging

## Abstract

Degenerative cervical myelopathy (DCM) is a common neurological condition, with disease progression that is both variable and difficult to predict. Here, we present a case of DCM in a gentleman in his late 60s with significant radiological disease progression without consequent change in clinical symptoms. The case serves as a reminder of an enduring medical aphorism that clinical history and examination should be prioritized above more complex data, such as imaging investigations. In addition, the case also highlights that guidelines should be contextualized within individual clinical circumstances.

## Introduction

Degenerative cervical myelopathy (DCM) is the most common cause of adult spinal cord dysfunction worldwide and is estimated to affect up to 2% of adults [1,2]. It arises secondary to degenerative pathology in the cervical spine, which leads to spinal cord compression. Cord compression may precipitate progressive neurological deficits including motor, sensory, and sphincter dysfunction [3].

The radiological progression of DCM does not always correlate well with its clinical progression [4-6]. Imaging findings such as the level and severity of spinal cord compression may not match the patient’s experience of their symptoms over time, thereby adding complexity to the clinician’s role of interpreting and reconciling clinical and radiological features of the disease [1,7].

Guidelines are clear in the event of symptom progression and advocate for surgery [8]. In contrast, the management of radiological progression without worsening symptoms is less defined and remains controversial. This report describes a case of the latter scenario, with the discussion focused on management.

## Case Presentation

A retired man in his late 60s presented reporting a 2-year history of lower limb weakness, impaired balance, and calf aches. He associated this with a preceding episode of influenza.

He had a background of long-standing lower back pain secondary to lumbar stenosis, for which he had previously undergone 3 surgical decompressions and atrial fibrillation, for which he took aspirin daily. His father had undergone a cervical laminectomy many years previously. The patient was otherwise fit and well, regularly cycled for exercise, and was a nonsmoker.

**Figure 1:**
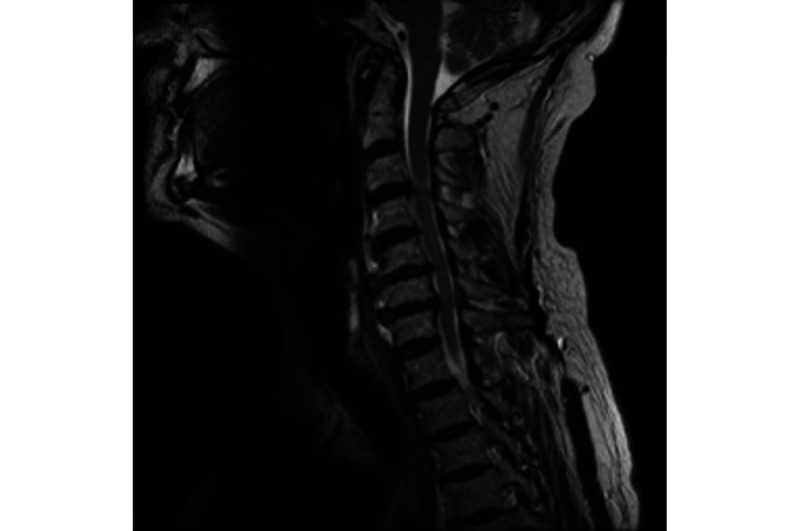
T2-weighted sagittal magnetic resonance imaging demonstrating multilevel degenerative cervical spondylosis and disc degeneration.

His symptoms resulted in a referral to neurology and investigations including magnetic resonance imaging (MRI) of his cervical spine. The MRI revealed multilevel degenerative changes from C3 to C7 in the cervical spine, on a background of a congenitally narrow spinal canal (Figure 1). He was therefore referred to the neurosurgery department. Consent was obtained from the patient.

On assessment in the neurosurgery clinic, the patient was diagnosed with DCM. His modified Japanese Orthopaedic Association (mJOA) score was 14 (4+6+2+2); a score of 12 to 14 indicates moderate myelopathy. Further exploration of his symptoms revealed dysesthesia in the region of the left shoulder, hypoesthesia of the third and fourth digits of the left hand, difficulties with tandem walking, and bladder issues with minor episodes of urinary incontinence. He was reluctant to undergo surgery, and hence, it was agreed to monitor his symptoms with reassessment in the clinic, with a strong recommendation to consider surgery if there was further progression.

**Figure 2:**
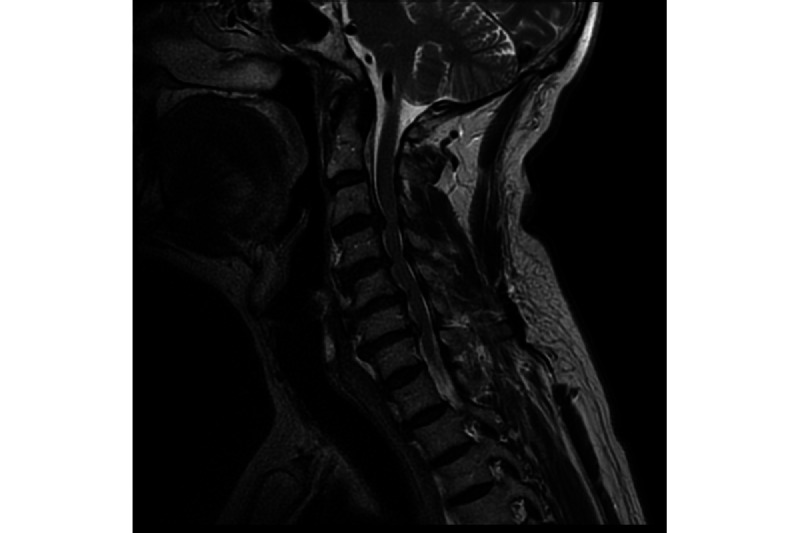
T2-weighted sagittal MRI demonstrating the progression of multilevel degenerative changes in the cervical spine, including disc degeneration at C3-C7.

The appointment 5 months later demonstrated no further progression of his symptoms, with an unchanged mJOA score. A further 6 months later, at a third neurosurgical clinic appointment, the patient reported some deterioration in his condition; he had been finding cycling more difficult, felt lower back stiffness, had worsened pain, and reported a mild electric shock sensation on the right upper limb. His mJOA score remained unchanged. He underwent a repeat MRI of his cervical spine, with a view to considering surgical decompression. The MRI showed progression of the degenerative changes at C3 to C7 (Figure 2).

Nonetheless, at his subsequent follow-up appointment, the patient reported improvement in the symptoms and the mJOA score remained 14. He had, however, developed left L4 sciatica, which had become his main concern. Three months later at his fifth appointment, his mJOA score was 13 (4+5+2+2). He reported increasing work and concentration required to walk and climb stairs. Compared to the third appointment, there had been a slow, gradual clinical progression. At this stage, the patient was put on the waiting list for surgery, just before the national lockdown in the United Kingdom during the COVID-19 pandemic.

**Figure 3:**
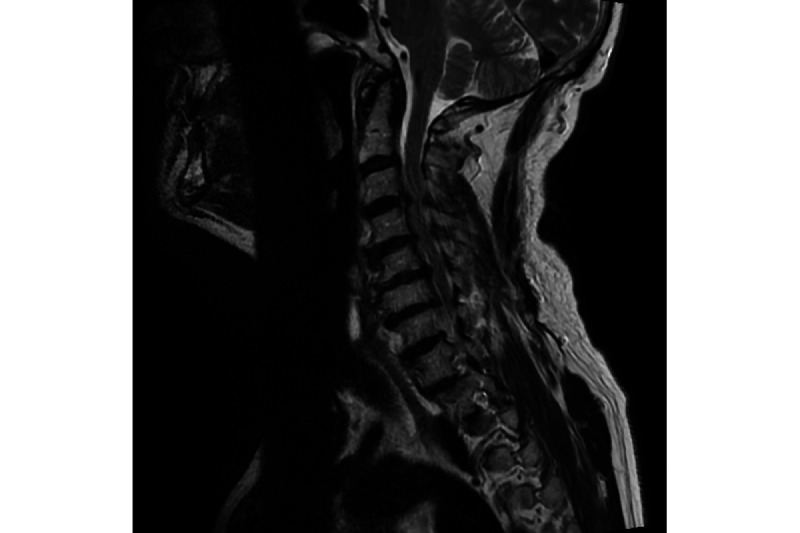
T2-weighted sagittal magnetic resonance imaging showing significant radiological worsening of cervical cord compression at C3-C7 levels despite unchanged symptoms and modified Japanese Orthopaedic Association clinical severity score.

At 3 subsequent follow-up appointments over the succeeding 8 months, there was no further clinical progression of the patient’s DCM. Therefore, it was agreed to remove him from the waiting list for surgery and continue with an expectant approach. At the ninth appointment 6 months later, symptoms were again stable. At the 10th appointment, the patient reported some worsening back and lower limb pain, as well as some deterioration in his general mobility. A further MRI was therefore requested, which demonstrated significant radiological progression of his DCM compared to his MRI 5 years earlier (Figure 3).

At the 11th appointment, 5 months later, the symptoms had improved to the patient’s previous baseline, including balance, dexterity, numbness, and urinary urgency. His mJOA remained 13. The radiological progression was in stark contrast with the fact that the patient remained clinically stable. Therefore, it was agreed to continue with expectant management and continue close clinical follow-up.

### Ethical Considerations

According to the National Health Service Health Research Authority, research ethics committee approval was not required for this work. Informed patient consent was obtained for publication.

### Investigations

The first MRI showed multilevel degenerative changes of the neck on the background of a congenitally narrow spinal canal: C3/4—disc osteophyte complex causing central compression of the spinal cord; C4/5—moderate stenosis; C5/6—moderate stenosis; C6/7—severe compression, with associated T2 signal changes.

The second MRI showed disc and facet joint degenerative changes between C3 and C7: C3/4—central disc protrusion causing moderate spinal canal stenosis and compression of the spinal cord, which had progressed compared to the first MRI; C4/5—broad-based disc bulge causing mild spinal canal narrowing and indenting the undersurface of the cord; C5/6—broad-based disc bulge causing moderate spinal canal narrowing and mildly compressing the spinal cord, which had progressed compared to the first MRI; C6/7—broad-based disc bulge causing mild to moderate spinal canal narrowing, indenting the anterior surface of the spinal cord.

The third MRI scan showed multilevel degenerative changes from C3 to C7, with scoliotic deformity: C3/4—severe circumferential stenosis with cord compression; C4/5—significant circumferential stenosis with cord compression; C5/6—circumferential stenosis with cord compression; C6/7—circumferential stenosis with cord compression.

### Differential Diagnosis

The differential diagnosis for a presentation of lower limb weakness, imbalance, and calf ache can be divided into upper and lower motor neuron patterns of weakness. Causes for the upper motor neuron pattern include pathologies in the brain and spinal cord. In the brain, this includes demyelinating disorders such as multiple sclerosis, vascular disorders such as stroke, space-occupying lesions such as a parasagittal meningioma or abscess, and motor neuron diseases such as amyotrophic lateral sclerosis. In the spinal cord, this includes demyelinating disorders such as transverse myelitis, myelopathies such as DCM, space-occupying lesions, for example, tumor or abscess, trauma, syringomyelia, and spinal stenosis (spinal claudication).

Causes for the lower motor neuron pattern include drugs such as alcohol, metabolic disorders such as vitamin B_12_ deficiency, diabetes mellitus, inherited disorders such as Charcot-Marie-Tooth, infections such as HIV or syphilis, and autoimmune disorders such as vasculitis and chronic inflammatory demyelinating polyneuropathy. The causes of calf aches include trauma, vascular disorders such as peripheral vascular disease (intermittent claudication), and inflammatory disorders such as myositis.

### Treatment

Since being diagnosed 5 years ago, an expectant approach has been taken in the management of this patient. International DCM management guidelines recommend surgical management for moderate, severe, or progressive DCM; however, for mild DCM, the optimal treatment strategy remains undefined, with a recommendation of either surgery or supervised nonsurgical management [8]. In this patient’s case, there is moderate DCM, with an mJOA score of 13, with significant radiological progression but clinical and symptomatic stability across serial assessments.

### Outcome and Follow-Up

The patient continues close neurosurgical follow-up, currently at 12 monthly intervals, alongside careful safety netting advice.

## Discussion

### Principal Findings

Degenerative changes in the cervical spine include disc herniation, osteophytosis, ligament hypertrophy, and ossification [9]. DCM is a clinical syndrome that arises when these changes result in spinal cord compression that is associated with symptoms, which may include neck pain or stiffness, limb pain or weakness, urinary incontinence, decreased manual dexterity, imbalance, or falls [1]. In this patient’s case, there was also a risk factor for congenital stenosis of the cervical canal.

A challenging aspect of the management of DCM is how to deal with symptoms changing over time and correlating this with evolving imaging findings. Another challenging aspect is the important decision on the timing of any surgical management.

The guidelines advise clinicians to take a structured, consistent approach to management. Each person with DCM requires consideration of individual factors, which may mean that clinical judgment or patient preferences result in deviation from guidelines in some circumstances. The patient was diagnosed with moderate DCM; the mJOA score for the patient was initially 14 and then decreased and remained stable at 13.

Strict application of the guidelines would lead to a recommendation for surgical intervention. However, while the mJOA includes consideration of upper and lower limb motor function, upper limb sensory function, and sphincter function, it does not capture all symptoms and clinical features. For example, in this case, limb pain was not captured. Nonetheless, it is a validated scoring tool for the assessment of functional status and is responsive to changes in the severity of DCM [10].

The complexity of this case requires a nuanced approach to management. Surgical intervention within DCM is primarily aimed at halting symptom progression; however, without symptom worsening, the decision of when to operate becomes more complicated. This patient’s symptoms were managed expectantly with nonsurgical interventions such as physical therapy as tolerated, oral analgesics, and neuropathic agents for any acute pain flares. Urinary symptoms were stable and were not actively managed. Waiting to operate at an older age may increase the risk of further complications, and this possibility should be explained to the patient. This should be part of a shared decision-making approach, where patients are empowered to make decisions through collaboration with their clinicians with the understanding that, in the context of a chronic disease like DCM, this decision will likely be revisited [11].

The factors driving the disconnect between clinical and radiological severity are unknown. Nonetheless, a model proposed by Davies et al [12] postulates that DCM is a function of (1) mechanical stress, (2) duration of injury, and (3) individual vulnerability_._ Using this model, a scenario of limited clinical progression and significant radiological progression over time could be explained by decreased vulnerability to injury.

An individual’s vulnerability to DCM comprises primary protective mechanisms such as genetics and age, in addition to adaptive protective mechanisms, such as autoregulation of spinal cord perfusion, functional reserve capacity, and nutritional status [12]. For example, certain genotypes are associated with increased regenerative capacity, such as the *HIF-1A* polymorphism rs11549467 [13]. This polymorphism is associated with susceptibility to DCM and its clinical features, including severity measured by mJOA.

Furthermore, adaptive protective mechanisms such as autoregulation of spinal cord perfusion may minimize ischemic injury. Decreased blood flow can result in blood-spinal cord barrier dysfunction, leading to microglia activation, neuroinflammation, and neuronal apoptosis [14]. In addition to the ischemia precipitating apoptosis [14], dysregulation of the autoregulatory system can occur from mechanical cord compression in DCM [15-17]. It is possible that dysregulation occurs to a lesser degree in some individuals, such as this patient. Furthermore, reserve capacity within the central nervous system [17-19] refers to resilience in the neurological system to account for the disconnect between the clinical phenotype and underlying histological pathology. In the context of DCM, cervical spinal cord compression and injury are initially asymptomatic, and the radiological changes affecting the spinal cord represent, at best, a proxy for the clinical presentation of the condition [12].

### Conclusions

In summary, individuals may have different vulnerabilities and protective mechanisms that may account for the disconnect between clinical and radiological features of DCM. The message is therefore to treat the patient rather than treating the findings from the imaging. This is especially important, given that asymptomatic cervical spinal cord compression is common in the general population [2,7].

## Abbreviations

DCM: degenerative cervical myelopathy
mJOA: modified Japanese Orthopaedic Association
MRI: magnetic resonance imaging

## Data Availability

All data generated or analyzed during this study are included in this published article.
